# A case of small-bowel and colon malformation identified during endoscopy in an adult patient

**DOI:** 10.1055/a-2216-0906

**Published:** 2023-12-08

**Authors:** Chen Wu, Xiangyu Liu, Lingyun Wang

**Affiliations:** 1 College of Clinical Medicine, Jining Medical University, Jining, China; 2 Department of Gastroenterology, Jining No. 1 People’s Hospital, Jining, China

A 57-year-old woman who had never undergone surgery previously was hospitalized due to a 6-month history of intermittent abdominal pain. Computed tomography revealed a communication between a portion of the small intestine and the adjacent segment of the sigmoid colon, with lumen dilatation and localized air and fluid accumulation ( Fig. 1 ).

**Fig. 1:**
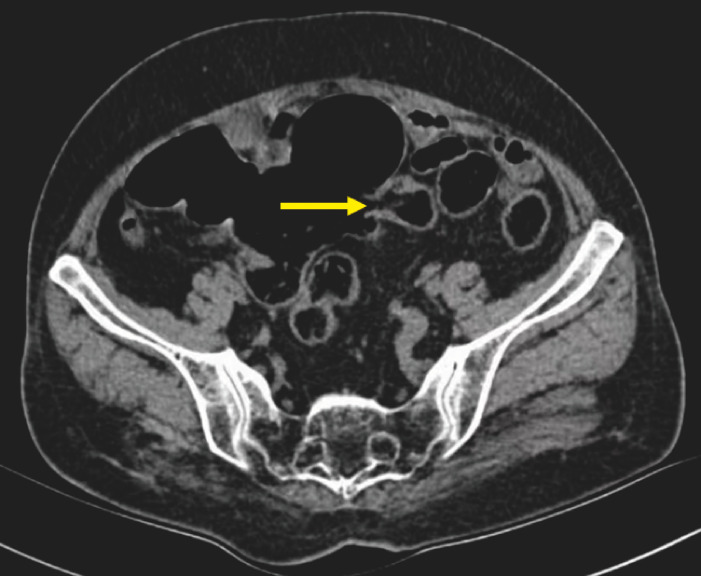
Computed tomography revealed a communication (arrow) between a portion of the small intestine and the adjacent segment of the sigmoid colon, with lumen dilatation and localized air and fluid accumulation.

The patient underwent gastroenteroscopy. Surprisingly, during colonoscopy, a fistula was observed 22 cm from the anus ( Fig. 2 ). After passing the scope through the fistula, three small intestinal lumens were detected ( Video 1 ), and a flat polyp measuring 1.2 × 0.8 cm in diameter was observed ( Fig. 3 ); therefore, a biopsy was performed. Attempts were made to explore the remainder of one of the small intestines up to 30 cm from the anus, but the blind end was not observed; similarly, resistance was encountered in the remaining two small intestines, preventing further advancement.

**Fig. 2:**
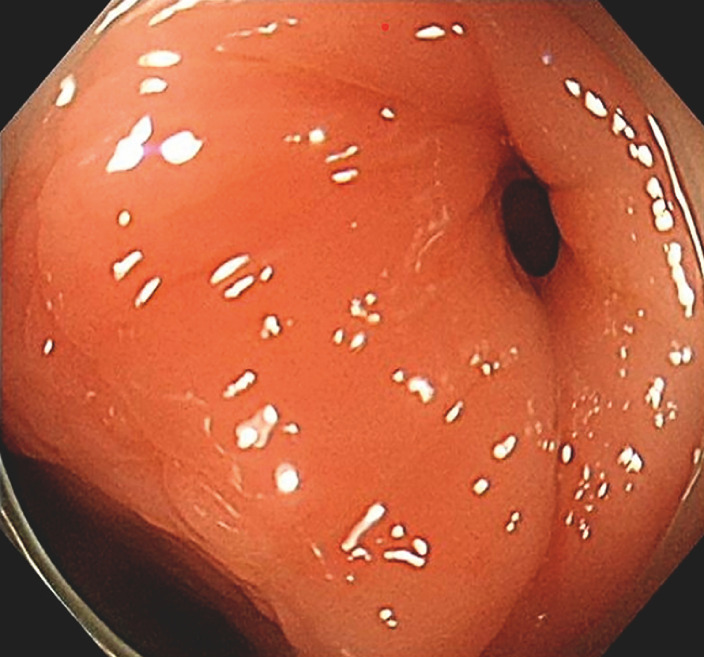
During colonoscopy, a fistula was observed 22 cm from the anus.

A case of small-bowel and colon malformation identified during endoscopy.Video 1

**Fig. 3:**
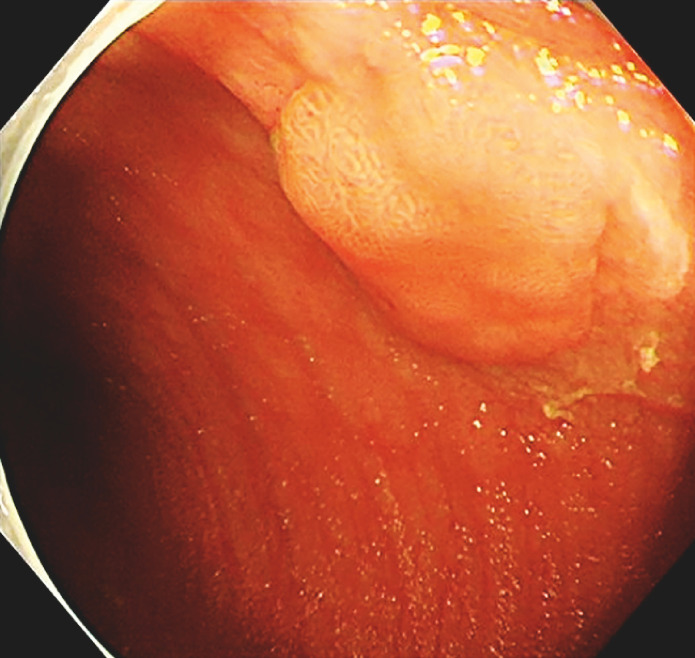
A flat polyp measuring 1.2×0.8 cm in diameter was observed.

Postoperative pathology indicated tubular adenoma with low grade intraepithelial neoplasia of the small intestine ( Fig. 4 ). The patient underwent small-bowel imaging after 1 day, which revealed a partial small-bowel dilatation ( Fig. 5 ). She refused surgical treatment and was discharged after being administered conservative treatment such as acid suppression and spasmolysis. Based on the abovementioned findings, the patient was diagnosed with malformation of the small bowel and colon, as well as tubular adenoma with low grade intraepithelial neoplasia.

**Fig. 4:**
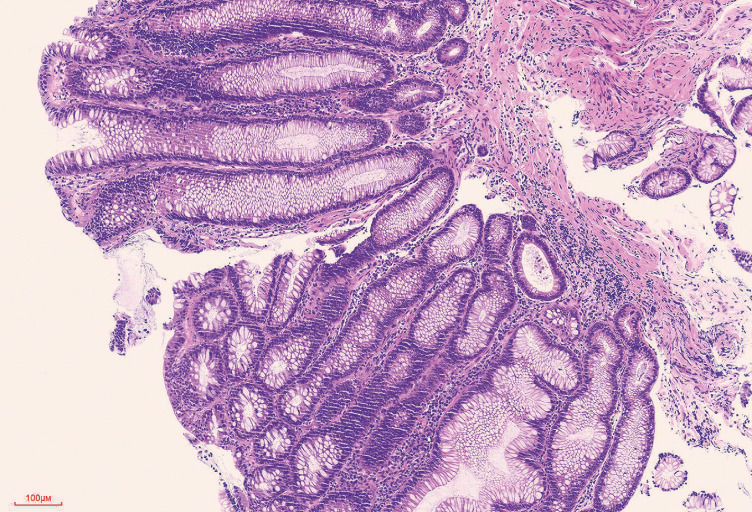
Microscopic examination: tubular glandular hyperplasia with mild atypia of adenoma cells (magnification ×10).

**Fig. 5:**
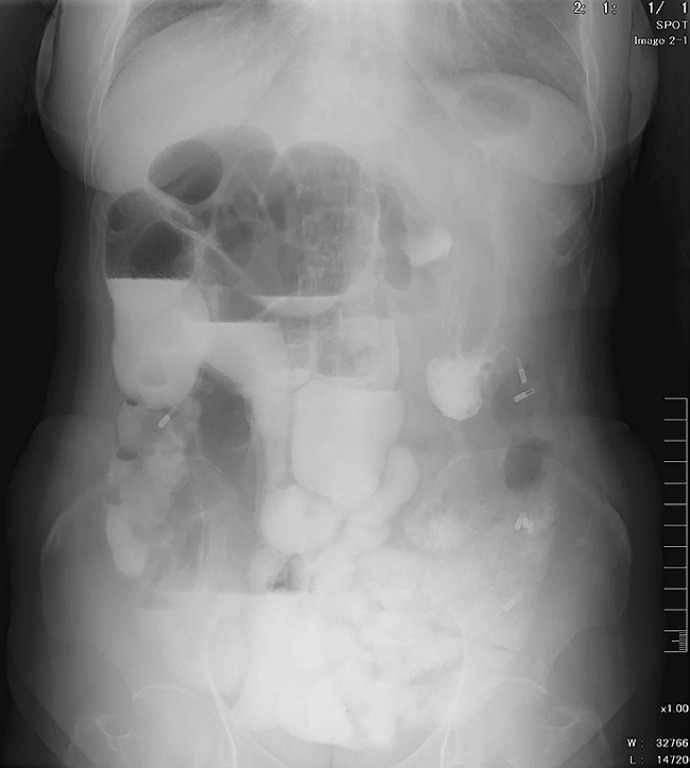
Small-bowel imaging revealed a partial small-bowel dilatation.

Gastrointestinal duplication is a rare congenital malformation that can involve any part of the gastrointestinal tract 1 . It prevalently occurs during infancy and childhood, and is rare in adults 2 . The clinical symptoms of adult gastrointestinal duplication are usually atypical, and imaging examinations are of limited diagnostic value. Generally, most of these malformations are diagnosed via surgical exploration, and only a few can be diagnosed by endoscopy 3 4 . Herein, we report a rare case of small-bowel duplication and colonic malformation in an adult, combined with tubular adenoma with low grade intraepithelial neoplasia, which provides experience for clinical workers to rationally apply auxiliary means of examination to avoid misdiagnosis.

Endoscopy_UCTN_Code_TTT_1AO_2AB

## Notes

Endoscopy E-Videos https://eref.thieme.de/e-videos
*E-Videos* is an open access online section of the journal *Endoscopy* , reporting on interesting cases and new techniques in gastroenterological endoscopy. All papers include a high-quality video and are published with a Creative Commons CC-BY license. Endoscopy E-Videos qualify for HINARI discounts and waivers and eligibility is automatically checked during the submission process. We grant 100% waivers to articles whose corresponding authors are based in Group A countries and 50% waivers to those who are based in Group B countries as classified by Research4Life (see: https://www.research4life.org/access/eligibility/ ). This section has its own submission website at https://mc.manuscriptcentral.com/e-videos .
